# Updated protocol: Effects of second responder programs on repeat incidents of family abuse: An updated systematic review and meta‐analysis

**DOI:** 10.1002/cl2.1200

**Published:** 2021-11-10

**Authors:** Robert C. Davis, Kevin Petersen, David Weisburd, Bruce Taylor

**Affiliations:** ^1^ National Police Foundation Arlington Virginia USA; ^2^ George Mason University Fairfax Virginia USA; ^3^ Hebrew University Jerusalem Israel; ^4^ NORC at the University of Chicago Bethesda Maryland USA

## Abstract

The US Department of Justice has extensively funded second responder programs. In England and Wales, funding of follow‐up with victims is largely funded by local Police and Crime Commissioners. While these programs rapidly gained popularity in the United States and are gaining popularity in other countries as well, the evidence regarding their effectiveness is mixed. Although some research has indicated that second responder programs can prevent repeat victimization, several experimental studies have suggested that these programs may actually increase the odds of abuse recurring. The purpose of the review is to compile and synthesize published and unpublished empirical studies of the effects of second responder programs on repeat incidents of family violence, including those studies completed after the original review. The Global Police Database (http://www.gpd.uq.edu.au/) provides a resource unavailable at the time of the initial review that will ensure that a comprehensive set of qualifying studies is identified. In the updated review, we will address the following questions: 1. What impact do second responder programs have on the number of subsequent calls to the police? 2. What impact do second responder programs have on abuse as measured on victim surveys? 3. Does the impact of second responder programs differ between experimental and quasi‐experimental studies or studies that employ different methods of drawing samples? Building on the original review, we also aim to expand our examination of effect size heterogeneity given sufficient data to do so. For instance, given the proposition that there may be only a small window of opportunity to intervene into the lives of family violence victims after an incident, the amount of time that elapses between a family violence call and the second response may be an important moderator of programmatic effects. Additional factors that could impact the effect of the intervention include the length of the follow‐up data collection period, the type of family violence complaint (e.g., intimate partner violence vs. elder abuse), and the sociodemographic characteristics of the victim and the offender (see generally Sherman, 2018). Ultimately, this review seeks not only to update the results of the prior review with additional research, but also to explore the mechanisms behind the observed effects in a way that provides utility for future policy creation.

## 1 BACKGROUND

### 1.1 The problem, condition, or issue

The literature on desistance of family violence suggests that the typical batterer's career is either short or sporadic: It has consistently been found that two in three households that report a domestic incident to the police do not report a subsequent incident over the following 6–12 months (see, e.g., Dowling & Morgan, 2019; Maxwell et al, 2010). But for those batterers who chronically abuse family members, it is no longer assumed that the initial police patrol response—especially those incidents where no arrest is made—is sufficient in and of itself to protect victims from recurrence of abuse. Experts have come to realize that legal sanctions or victim actions that raise the personal or social costs to the batterer may promote a reduction or cessation in abuse (Fagan, 1989; Felson et al., 2005). Effective solutions to family violence (including intimate partner abuse, abuse within families or households, and elder abuse) must involve efforts to educate victims about their options and connect them with counseling, relocation, civil legal assistance, and other services that can lessen dependence on the abuser.

### 1.2 The intervention

During the 1980s a program model was developed in which social workers (“second responders”) visit homes in which family violence incidents were recently reported to the police to help them find long‐term solutions to recurring abuse (e.g., see Dean et al., 2000; Mickish, 2002). Second responder programs are based on the premises that family violence often recurs and that victims are likely to be especially receptive to crime prevention opportunities immediately following victimization. That is, there is a “window of opportunity” during the first few hours or days after a crime during which victims feel vulnerable and are willing to seriously consider behavioral and lifestyle changes (Anderson et al., 1995; Davis & Smith, 1994; see also Scott et al., 2015). In second response programs, a team, usually consisting of a police officer and a social worker, follow‐up on the initial police response to a family violence complaint. The team provides the victim with support in accessing services such as housing, legal assistance, and safety planning. The team may provide information on these services and inform victims of their legal options. The team may assist the victim by providing referrals, facilitating an initial contact with a service provider, helping the victim to complete applications, and advocating on the victim's behalf. In some models, the team also may warn those perpetrators present at the follow‐up of the legal consequences of continued abuse.

### 1.3 How the intervention might work

The purpose of working directly with the victims is to reduce the likelihood of a new offense by helping them to understand the cyclical nature of family violence, develop a safety plan, obtain a restraining order, increase their knowledge about legal rights and options, and provide shelter placement or other relocation assistance. In other words, second responder programs seek to “empower victims to access social service, mental health, and advocacy measures, so that they can take action to reduce their risk for future victimization” (Scott et al., 2015, p. 276). A secondary aim of the intervention with victims may be to establish greater independence for victims through counseling, job training, public assistance, or other social service referrals. The purpose of conversations with abusers is to ensure that they understand that assaulting an intimate is criminal and that further abuse will result in (additional) sanctions.

Koppensteiner et al. (2019) view the role of second response teams as lowering the cost to victims of accessing support services. They argue that second responder programs remove barriers that impede victims from accessing services. These barriers may include lack of knowledge about service program eligibility, application procedures, hours of operation, and so forth. They may also originate from victims speaking a different language or having low self‐efficacy due to depression or substance abuse issues. Fugate et al. (2005) found that these perceived barriers were important reasons why victims failed to access support services.

It could be argued that second responder programs might affect recidivism through several channels. The second responders, or the service programs that they encourage victims to access, could persuade victims to end their abusive relationship and/or separate from the abuser. Alternatively, the services and educational resources provided to victims may enable them to better navigate and mitigate the risks of victimization within their relationships. These programs may also exert a deterrent effect on abusers directly through warnings or indirectly by encouraging and supporting victims in the prosecution process.

### 1.4 Why it is important to do the review

A series of field tests carried out in New York (Davis & Medina, 2001; Davis & Taylor, 1997; Taylor, n.d.) suggested a possible iatrogenic effect of a second response program. A pooled analysis conducted by Davis et al. (2006) reanalyzed data from three separate field experiments, each testing the same intervention on somewhat different populations. The pooled analyses indicated that the interventions were associated with an increase in reporting of new abusive incidents not only to authorities (which could indicate simply greater confidence in the police), but also to research interviewers. The New York field tests suggested that second response programs might actually increase the likelihood of new abuse. It is not unheard of for criminal justice programs to produce iatrogenic effects (e.g., Braga & Weisburd, 2012). Given the potential for interventions to cause harm if the design is flawed, it is important to verify empirically that the logic model is correct, and that the program really does have the benefits that were intended.

Other work, however, suggested that second responder programs are effective in reducing subsequent family abuse. A quasi‐experiment by Greenspan et al. (2003) found that victims who received a second response were less likely to report victimization on a subsequent survey. An experiment by Pate et al. (1992) also found a decrease in subsequent violence reported on a survey following a second response.

The 2006 review yielded inconclusive finding about second responder programs. The review examined two outcomes: further reports by the victim of domestic abuse to the police and reports of abuse by the victim in response to a research survey. The latter measure could be expected to be a purer measure of abuse since reports to the police may be partially dependent on victims' confidence in the police. A rapid literature review by Mazerolle et al. (2018, p. 30) concluded that:

….perceptions of and experiences with police directly influence victim's willingness to report. A perceived inadequate response by police in the past…can make victims reluctant to report re‐victimisation.

The 2006 meta‐analysis found a small effect of the intervention on victim reports to the police, but no effect on reports of abuse on research surveys. One interpretation might be that the second responder intervention increases victim confidence in the police but does not reduce the true prevalence of abuse. However, the results do not give a clear indication whether this type of intervention merits public funding.

The model has continued to be adopted by police agencies in the United States, with New York City recently expanding a pilot program in five precincts to all 76 New York Police Department (NYPD) precincts.^1^ A preliminary search turned up several new evaluations of second responder programs with stronger indications of program impact than the set of studies included in the initial review. Both of these facts argue for a re‐examination of the intervention. Second responder programs have also been implemented in other countries. Silke (2011) reports on the creation of the PRADO (Partnership Responses at Domestic Violence Occurrence) program in Australia, now expanded to several additional sites. Reports on second responder programs have also been issued in Canada (Scott et al., 2015) and the UK (Koppensteiner et al., 2019).

Finally, the initial review did not examine differences in the experimental study methodologies that might have affected findings. Studies have used different approaches to gathering samples. One approach used, for example, by Koppensteiner et el. (2019) is to randomly assign cases as they are reported to the police to either a second response or a control condition. However, nearly half of the victims assigned to the second response group failed to engage (i.e., refused the service or could not be reached). The other used, for example, by Stover et al. (2010) is to contact victims in eligible cases and recruit them into the study before the random assignment process. In the Stover et al. study, 25% of the initial pool of victims agreed to participate. One approach raises concerns about attrition from treatment and the other concerns about generalizability. An updated review would examine experimental study outcomes as a function of study designs.

## 2 OBJECTIVES

The US Department of Justice has extensively funded second responder programs. In England and Wales, funding of follow‐up with victims is largely funded by local Police and Crime Commissioners. While these programs rapidly gained popularity in the United States and are gaining popularity in other countries as well (e.g., Smee, 2021), the evidence regarding their effectiveness is mixed. Although some research has indicated that second responder programs can prevent repeat victimization, several experimental studies have suggested that these programs may actually increase the odds of abuse recurring.

The purpose of the review is to compile and synthesize published and unpublished empirical studies of the effects of second responder programs on repeat incidents of family violence, including those studies completed after the original review. The Global Police Database (GPD; http://www.gpd.uq.edu.au/) provides a resource unavailable at the time of the initial review that will ensure that a comprehensive set of qualifying studies is identified. In the updated review, we will address the following questions:

1. What impact do second responder programs have on the number of subsequent calls to the police?
2. What impact do second responder programs have on abuse as measured on victim surveys?
3. Does the impact of second responder programs differ between experimental and quasi‐experimental studies or studies that employ different methods of drawing samples?

Building on the original review, we also aim to expand our examination of effect size heterogeneity given sufficient data to do so. For instance, given the proposition that there may be only a small window of opportunity to intervene into the lives of family violence victims after an incident (Anderson et al., 1995; Davis & Smith, 1994), the amount of time that elapses between a family violence call and the second response may be an important moderator of programmatic effects. Additional factors that could impact the effect of the intervention include the length of the follow‐up data collection period, the type of family violence complaint (e.g., intimate partner violence vs. elder abuse), and the sociodemographic characteristics of the victim and the offender (see generally Sherman, 2018). Ultimately, this review seeks not only to update the results of the prior review with additional research, but also to explore the mechanisms behind the observed effects in a way that provides utility for future policy creation.

## 3 METHODS

### 3.1 Criteria for considering studies for this review

#### 3.1.1 Types of studies

The scope of this review is experimental and nonequivalent control group quasi‐experimental studies. To be considered eligible, studies must include a comparison group which did not receive a second response and must report on at least one measure of repeat family violence taken during the postintervention period. The comparison group must consist of cases meeting the same criteria as treatment group cases (i.e., domestic violence complaints in both groups, elder abuse complaints in both groups, general family abuse complaints in both groups, etc.) and must include either the same victim, the same victim/offender pairing, or the same offender. Comparison groups may be drawn from the same geographic area (e.g., police precinct) as the treatment group, from another geographic area identified as having a similar case and demographic make‐up, or from an earlier (preintervention) time period.^2^

Control groups may be matched using simple comparisons of descriptive statistics, statistical tests of baseline characteristics, or statistical matching techniques (e.g., propensity score matching). Matching of treatment and control groups will not be a requirement for study eligibility, however, quasi‐experimental studies that do not match treatment and control groups must attempt to limit the influence of confounding factors (e.g., multiple regression with covariates, ANCOVA, etc.). In other words, we will include unmatched control group designs so long as the comparison group has either face validity or the researchers attempt to statistically limit one or more factors that may influence the outcome. We will distinguish between matched and unmatched groups in a moderator analysis. Additionally, because we anticipate that the primary unit of analysis will be victims or households (e.g., victim/offender pairings), it is unlikely that many studies will have preintervention measures of victimization. As such, eligible studies will only be required to report postintervention outcome measures.

Eligible studies must meet the methodological criterion used for inclusion in the GPD (http://www.gpd.uq.edu.au/). The GPD will be the primary search source for this review, however, only the following subset of research designs adapted from the Global Policing Database protocol will be considered eligible (see Higginson et al., 2015, pp. 47–48):

- Randomized experimental designs (RCT)
- Regression discontinuity designs
- Matched control group designs with or without preintervention baseline outcome measures (statistical or propensity score matching)
- Matched control group designs with preintervention measures of descriptive statistics (comparison of baseline characteristics)
- Short interrupted time‐series designs with control group (<25 pre and 25 postintervention observations [Glass, 1997])
- Long interrupted time‐series designs with control group (≥25 pre‐ and postintervention observations ([Glass, 1997])
- Unmatched control group designs where the control group has face validity
- Unmatched control group designs analyzed with covariates.

### 3.2 Types of participants

The experimental units must be either victims for survey outcome measures or households (street addresses) for repeat calls for police service. Our primary interest concerns the impact of second responder programs on repeat victimization within these experimental units. As such, our populations of interest include family violence victims of all demographic characteristics (i.e., any race, age, sex, etc.), family violence offenders of all demographic characteristics, and households in which family violence has occurred (to include any address where family violence victims and offenders reside).

### 3.3 Types of interventions

Included studies must be evaluations of a second responder program; that is a program operated by or in cooperation with a municipal law enforcement agency in which, in response to a family violence complaint (complaints involving intimate partners, family members, or persons cohabiting), the police summon a family violence specialist or specialists to visit victims at their homes or police station. These specialists could be victim advocates and/or specially trained police officers. The content of the contact must be aimed at reducing the likelihood of a repeat offense and providing or connecting the victim with social services. It could include information about the nature of family violence, safety planning, information about legal rights and services, shelter placement, relocation assistance, and referrals to social services. We will not include those programs that contact victims only by mail or phone.

### 3.4 Types of outcome measures

Two main outcome measures will be considered for this review:

1. Repeat family violence/victimization based on police data (official data)
2. Repeat family violence/victimization based on survey or interview data (unofficial data).

Repeat violence based on official data is concerned with whether any new family violence incidents were reported to the police in the form of calls for service or crime reports during the postintervention follow‐up period. This outcome may be either a continuous or dichotomous measure indicating the number of (or presence/absence of) family violence incidents reported to the police after the second response intervention that occurred within the same household, victim, or victim‐offender pairing as the triggering offense. Family violence complaints may include domestic/intimate partner violence, elder abuse, or general/aggregate measures of family violence.

Repeat violence based on unofficial data will likely be measured through victimization surveys or victim interviews conducted by researchers or study authors during the postintervention period. This outcome is also expected to be either a continuous or dichotomous measure of repeat incidents occurring within the same household, victim, or victim‐offender pairing but is not limited to offenses that were officially reported to the police. Self‐report outcomes may also include domestic/intimate partner violence, elder abuse, or general/aggregate measures of family violence. If studies contain additional measures, such as increased victim use of social services or increased confidence in the police resulting from the intervention, we will make note of these outcomes. But we do not expect to find enough studies incorporating these outcomes to warrant a dedicated analysis.

Eligible studies must include at least one of the above outcomes and employ an appropriate methodological design (as discussed above). Distinguishing these outcomes by their form of measurement is an important aspect of this review. As the results of the initial review suggested, reports of new abuse made to the police may be ambiguous as to any true programmatic effect when examined in isolation. This is because an increase in reports may indicate either an increase in abuse or no change in abuse but greater confidence in the police as a result of the second responder intervention. Therefore, we will be especially interested in studies that include reports of new abuse obtained from victim surveys, a more clear‐cut measure of new abuse.

### 3.5 Duration of follow‐up

We will not restrict eligibility to any particular follow‐up period. Studies may vary in their length of follow‐up, however, based on the studies included in the initial review we expect that most studies will have follow up periods of either 6 or 12 months. Should we find notable variation in follow‐up length across our eligible studies we will examine the effect of follow‐up length on effect size. To do so we will conduct a regression‐based moderator analysis using follow‐up length as a continuous independent variable (see Higgins et al., 2020; Viechtbauer, 2010). If studies report multiple follow‐up periods, we will separate results into discrete follow‐up categories to ensure that each study is represented only once in each analysis (i.e., 6 months or less, 6–12 months, greater than 12 months, etc.).

### 3.6 Types of settings

Eligible interventions must target second responder programs at family violence victims, victim‐offender pairings, or residences/addresses. There will be no restriction on settings within these categories. Victims, offenders, and residences may come from any geographic, ethnic, cultural, or demographic makeup so long as the study otherwise fits our inclusion criteria. There will be no language or publication restriction imposed on our eligible studies. For non‐English language studies we will use Google Translate to screen titles and abstracts. We will also use Google Translate to screen any non‐English language titles that require full‐text review. Where the above strategies do not result in a clear eligibility determination, we will contact study authors to verify eligibility. Studies archived between 1970 and 2018 (or the most recent year of GPD data available at the time of the data extraction) will be included in this review.

### 3.7 Search methods for identification of studies

#### 3.7.1 Electronic searches

The search for this updated review will be led by the GPD research team at the University of Queensland (Elizabeth Eggins and Lorraine Mazerolle) and Queensland University of Technology (Angela Higginson). The University of Queensland is home to the GPD (see http://www.gpd.uq.edu.au), which will serve as the main search location for this review. The GPD is a web‐based and searchable database designed to capture all published and unpublished experimental and quasi‐experimental evaluations of policing interventions conducted since 1950. There are no restrictions on the type of policing technique, type of outcome measure or language of the research (Higginson et al., 2015). The GPD is compiled using systematic search and screening techniques, which are reported in Higginson et al. (2015) and summarized in Supporting Information Appendices A and B. Broadly, the GPD search protocol includes an extensive range of search locations to ensure that both published and unpublished research is captured across criminology and allied disciplines.

The following search string will be used to search the title and abstract fields of the GPD corpus of full‐text documents that have been screened as reporting on a quantitative impact evaluation of a policing intervention. These terms will be used to capture studies published from 1970 to 2018. Here, “TI” represents keyword combinations that are limited to title fields and “AB” represents keyword combinations limited to abstract fields:

1. (TI: (“second respon*” OR coordinate* OR multiagency OR integrated OR visit* OR service* OR interven* OR program* OR advocate* OR “social work*” OR counsel* OR psychologist* OR health* OR clinician*)) OR (AB: (“second respon*” OR coordinate* OR multiagency OR integrated OR visit* OR service* OR interven* OR program* OR advocate* OR “social work*” OR counsel* OR psychologist* OR health* OR clinician*))
  AND
2. (TI: (domestic* OR wife OR wives OR husband* OR partner* OR intimate* OR relationship* OR family OR familial OR families)) OR (AB: (domestic* OR wife OR wives OR husband* OR partner* OR intimate* OR relationship* OR family OR familial OR families))
  AND
3. (TI: (abuse* OR aggress* OR assault* OR batter* OR coercive* OR chok* OR death* OR beat* OR harm* OR femicide* OR homicid* OR infanticide OR lethal* OR murder* OR manslaughter* OR injur* OR shoot* OR stab OR stabb* OR strangl* OR strangul* OR violen* OR weapon*)) OR (AB: (abuse* OR aggress* OR assault* OR batter* OR coercive* OR chok* OR death* OR beat* OR harm* OR femicide* OR homicid* OR infanticide OR lethal* OR murder* OR manslaughter* OR injur* OR shoot* OR stab OR stabb* OR strangl* OR strangul* OR violen* OR weapon*))

After conducting the initial GPD search, we will search additional gray literature repositories that are focused on domestic and family violence outside of the United States. These repositories will include the following:

- Queensland Center for Domestic and Family Violence Resources (https://noviolence.org.au/)
- Australia's National Research Organization for Women's Safety (https://www.anrows.org.au/)
- Stopping Family Violence (https://sfv.org.au/)
- Domestic Violence Prevention Centre (https://domesticviolence.com.au/)
- SaveLives (https://safelives.org.uk/about-us)
- Women's Aid (https://www.womensaid.org.uk/)
- Women Against Violence Europe (https://www.wave-network.org/)
- The Federal Ministry for Family Affairs, Senior Citizens, Women and Youth (https://www.bundesregierung.de/breg-en/federal-government/ministries/ministry-of-family-affairs).

#### 3.7.2 Searching other sources

We will supplement the results of the GPD search with several additional search strategies. First, we will examine the reference sections of all studies determined to be eligible for mention of additional studies that are potentially relevant, as well as any prior reviews that are identified. Second, we will search 2020 and 2021 volumes of leading journals in the field to identify any recent studies that may not yet be indexed in the GPD, including *Police Quarterly, Policing and Society, Policing, Police Practice and Research, Violence against Women, Journal of Interpersonal Violence, Journal of Family Violence, Violence and Victims*, and *Criminology and Public Policy*. Third, after finishing the above searches and reviewing the studies as described later, we will e‐mail the list to authors of the qualifying studies to ask them to identify eligible studies that do not appear on our list, particularly dissertations and other unpublished research reports. Finally, we will search the US Office of Violence Against Women (https://www.justice.gov/ovw) website for a listing of federally funded second responder programs and any evaluations conducted on those programs.

We will seek to obtain full text versions of potentially relevant studies using the George Mason University library. We will seek to obtain interlibrary loans for any paper not available directly from the George Mason library. If there are papers that cannot be obtained through these libraries, we will contact the study authors to request a copy of the full‐text version of the study.

### 3.8 Data collection and analysis

#### 3.8.1 Description of methods used in primary research

Eligible studies for this review will be experimental or quasi‐experimental impact evaluations that include a treatment group (group receiving a second response), a control/comparison group (group not receiving a second response), and provide an outcome measure of repeat family violence taken during the postintervention period. As an example, Davis and Taylor (1997) evaluated a second response intervention conducted in collaboration between the NYPD and New York Victim Services. Family violence calls for service were randomly assigned to receive either a second response or no second response. In the treatment condition, teams of police officers and social service workers provided in‐person follow‐up to family violence complaints within 7–14 days of the call. To evaluate the intervention, Davis and Taylor examined repeat family violence calls for service and administered victimization surveys six months after the second response.

In the majority of studies, we anticipate that the unit of analysis will be the household or the victim‐offender pairing. At times, however, the unit of analysis may be the victim, regardless of whether they remained at the same address or in a relationship with the initial offender. Judging from the initial review, we expect an approximately even number of experimental and quasi‐experimental designs. Additionally, while the majority of studies are likely to report outcome measures at 6‐ or 12‐month follow‐up periods, some studies may provide longer follow‐up periods or multiple follow‐up periods within the same study.

### 3.9 Criteria for determination of independent findings

Each study (here defined as a unique research sample) will be included no more than once in each distinct analysis. Where multiple papers report on the same underlying intervention, we will code all reports and choose the one deemed most complete from which to retrieve data (though information may be extracted from multiple reports when necessary or to provide more comprehensive coding). Where a study or multiple reports concerning the same intervention contain multiple measurement time‐points, each will be coded and synthesized separately based on the length of follow‐up period (e.g., short, medium, and long). Given a sufficient number of studies in each of these categories, we will conduct separate analyses for each follow‐up category. If few studies report multiple follow‐up periods, we will select the follow‐up period that is most homogenous with our remaining studies, thus ensuring that each study is represented only once in each analysis. If there are multiple jurisdictions included in a single study (i.e., a multisite trial), each site will be considered a separate study, so long as each site has a unique control group.

While we do not anticipate clustered designs (e.g., group assignment to second responder intervention rather than individual assignment), we will deal with hierarchical data structures in multiple ways. If the study accounts for clustering in their statistical analysis (e.g., multilevel models or clustered standard errors), we will attempt to directly use the adjusted estimates that are provided to obtain the effect size. If clustering is not accounted for in the statistical analysis but an intra‐class correlation coefficient is provided (ICC), we will use the method described by Fu et al. (2013) to adjust the standard error of the effect sizes. If no ICC value is provided, we will conduct sensitivity analyses at various ICC values (see Armstrong et al., 2018).

It is also possible for studies to report multiple outcomes from within the same outcome grouping (i.e., official measures of repeat family violence and unofficial measures of repeat family violence) and the same research sample. For instance, a study could report repeat domestic abuse and repeat elder abuse complaints that are both measured with official police data or multiple forms of repeat abuse measured through victim surveys. To the extent possible, we will separate these distinct outcomes into separate statistical analyses. For instance, while we plan to estimate models of repeat abuse using official data separately from models of repeat abuse using unofficial data, we will also estimate models of repeat domestic abuse using official data separately from models of repeat elder abuse using official data, assuming a sufficient number of studies report both. If very few studies report multiple outcomes, however, we will first attempt to select the outcome that is most homogenous with our remaining studies. If such a selection cannot clearly be made but the study provides raw data that can be aggregated (e.g., bivariate frequencies/proportions or raw counts), we will calculate composite effect sizes by combining the raw data from each outcome. If data cannot be combined, we will apply selection rules to prioritize certain outcomes over others. That is, we will prioritize domestic violence over elder abuse, physical abuse over psychological abuse, total number of reported incidents over incident severity (e.g., as measured by instruments such as the conflict tactics scale or individual components of this scale), victim survey measures over offender survey measures, and repeat incidents involving the same victim/offender pairing over repeat incidents involving the same victim alone.

If none of the above selection rules can be applied and only a small number of studies report multiple outcomes from within the same outcome grouping (e.g., official vs. unofficial measures), we will simply average the effect sizes and standard errors for the few studies that report multiple outcomes. Lastly, if a significant number of studies report multiple outcomes from within the same outcome grouping that cannot be separated into discrete analyses or reduced through the above selection rules, we will employ robust variance estimation to utilize all effect sizes simultaneously (Hedges et al., 2010). In other words, if a large proportion of studies report multiple measures of repeat family violence using official data or multiple measures of repeat family violence using unofficial data that cannot be handled with the above strategies, robust variance estimation would be used to combine effect sizes for each outcome grouping (official and unofficial data sources) within each study. Separate models for official and unofficial measures of repeat family violence would then be estimated using these combined effect sizes.

### 3.10 Selection of studies

Potential candidates found through the search procedures described above will be examined for relevance by the search team. As a first step, all screeners will review the titles and abstracts of the same set of 25 results to establish reliability. Any discrepancies in this pilot screening stage will be discussed among the research team. The remaining results will then be divided between screeners and assessed for their potential relevance to second responder programs. All abstracts will be coded as “relevant,” “not relevant,” or “maybe.” Any results coded as “maybe” will be discussed among the research team before making a final determination. Results that are clearly ineligible but may be useful for narrative review will be marked as such.

All titles/abstracts deemed “relevant” during initial screening will proceed to full‐text review. As a first step, all screeners will review the full‐text versions of the same set of 25 results to establish reliability. Any discrepancies in full‐text eligibility will be discussed among the research team before proceeding with full‐text review. All remaining studies will be divided among screeners and independently assessed for both relevance to second responder programs and methodological eligibility. At this stage, all results will be marked as “eligible,” “ineligible,” or “unsure.” All results marked as “unsure” will be discussed among the research team. Any results that are clearly ineligible but may be relevant for review will be marked as “relevant for review” at this stage.

### 3.11 Data extraction and management

All studies deemed eligible after title/abstract review and full‐text review will be double coded by Kevin Petersen (an author of this protocol) and another study author. A detailed coding protocol will be used to extract as much pertinent information for analysis as possible from each report or article (see Supporting Information Appendix C). Coders will compare coding results for each study and any uncertainty or disagreement between coders will be resolved through discussion and consultation. Any remaining disputed cases will be resolved by a third principal member of the research team.

All studies will be coded across a variety of study‐level, outcome‐level, and effect size‐level measures (see Supporting Information Appendix C), including but not limited to:

A) Reference information (publication type, research team, etc.)
B) Description of the study site and intervention (geographic area, nature of the second response, dosage and intensity, etc.)
C) Methodology (assignment process, nature of treatment and control groups, sample sizes, etc.)
D) Implementation issues (risk of bias, threats to internal validity, etc.)
E) Results (raw data, statistical significance, etc.)
F) Conclusions reached by the authors.

### 3.12 Assessment of risk of bias in included studies

Studies deemed eligible for inclusion in the meta‐analysis will be assessed for risk of bias using the Cochrane randomized and nonrandomized risk of bias tools, based on study design (Higgins et al., 2019). Coding items consistent with these tools will allow for the categorization of randomized experiments into low risk, some concerns, or high‐risk classifications, and quasi‐experimental designs into low risk, moderate risk, serious risk, or critical risk classifications. Adaptation of these risk of bias tools may be necessary to better fit the issues prevalent in our eligible studies. Any such modifications will be made during the course of the review as prevalent issues become more apparent. Risk of bias ratings across eligible studies will be presented in tabular and narrative format and sensitivity analyses may be performed depending on the level of bias noted in our eligible studies.

### 3.13 Measures of treatment effect

Based on the prior review, it is anticipated that the primary unit of analysis will be at the individual, pair, or household level. Given that the number of repeat offenses that occur during postintervention follow‐up periods may often be small, we anticipate that some studies will report their results using means and standard deviations while others will report their results using bivariate frequencies/proportions (i.e., proportion of cases with no repeat violence vs. proportion of cases with repeat violence). Where studies report their results using means and standard deviations, we will calculate Cohen's *d* values, and when possible, will apply the small sample size correction to convert these values into Hedge's *g* effect sizes. Where studies report their results using bivariate frequencies or proportions, we will calculate logged odds ratios (Lipsey & Wilson, 2001). In either case, it is likely that the effect sizes in some studies will require conversion to present all results in a common effect size metric. Given that conversions from odds ratios to Cohen's *d* values (and vice‐versa) are only approximations and can be biased (see Sánchez‐Meca et al., 2003), our primary effect size metric will be the one that requires the least number of conversions. Whether converting from an odds ratio to a Cohen's *d* value or vice versa, we will use the Cox logit method for all conversions (see Sánchez‐Meca et al., 2003).

Should a study provide data for the calculation of both a Cohen's *d* value and a logged odds ratio, we will prioritize the effect size metric that is most homogeneous with our remaining studies. If a significant number of studies provide both forms of data, we will choose the metric that best represents the distribution of the outcome measure. In other words, if the number of repeat incidents is generally low with a notable number of cases that did not experience any repeat victimization, we will prioritize odds ratios as the effect size metric. However, if the number of repeat incidents is moderate to high and/or few cases did not experience any repeat victimization, we will prioritize Cohen's *d* measures of effect size. Lastly, while we do not anticipate that studies will use geographic areas as units of analysis, we will calculate incident rate ratios or relative incident rate ratios for any studies that report counts of repeat violence within a geographic area and risk ratios for any studies that report dichotomous outcomes within a geographic area (see Wilson, 2021).

### 3.14 Dealing with missing data

In situations where eligible studies are missing the requisite data to calculate effect sizes or make other necessary coding determinations, we will contact study authors to try and obtain the information needed. If the information cannot be obtained, these studies will be excluded from the meta‐analysis but included in our narrative review to limit reporting bias.

### 3.15 Assessment of heterogeneity

The total amount of effect size heterogeneity in our analyses will be assessed using the *Q* statistic and related significance test. However, a nonsignificant *Q* statistic will not be interpreted as a lack of excess heterogeneity. Rather, we will assume a random effects model and report the amount of between‐study variance in effect sizes using the *τ* ^2^ statistic. Lastly, the proportion of the total variance in effect sizes attributable to heterogeneity across studies will be measured using the $I^{2}$ statistic.

### 3.16 Assessment of reporting biases

We will use four approaches to assess reporting bias in our meta‐analyses. First, we will conduct simple comparisons of mean effect sizes for published and unpublished studies. Second, we will generate a funnel plot and visually inspect it for signs of asymmetry. Third, we will conduct a trim‐and‐fill analysis. If asymmetry is detected, we will compare the mean effect size of the plot after symmetry is achieved (through the imputation of additional effect sizes) with the mean effect size of the original plot (Duval & Tweedie, 2000). Lastly, we will conduct an Egger's regression test for funnel plot asymmetry (Egger et al., 1997).

### 3.17 Data synthesis

Effect sizes will be synthesized using inverse‐variance weighted meta‐analysis. It is unlikely that the impact of second responder programs, which may vary in nature, would share a common effect across units of analysis that differ on demographic, geographic, and socioeconomic characteristics. Thus, we will assume a random effects model for all analyses. Such an assumption is now considered best practice in many fields, given that the *Q* statistic is often underpowered and that the random effects model will converge on the fixed‐effects model in the absence of excess heterogeneity (Borenstein et al., 2010). Random effects models will use restricted maximum likelihood estimation, and all data synthesis will be conducted using R statistical software and the *metafor* package (Viechtbauer, 2010).

### 3.18 Subgroup analysis and investigation of heterogeneity

We plan to employ several moderators to investigate potential sources of effect size heterogeneity. The moderating variables that can be examined will be dependent on the results of our systematic search strategies, however, potential moderators include research design (also a test for publication bias), sampling approach (volunteer vs. nonvolunteer), type of family violence complaint (e.g., intimate partner violence vs. elder abuse), length of data collection follow‐up period (measured as a continuous variable and/or a categorical variable), and length of time between the initial police call and the second response contact (responses closer in time to the triggering incident may better capitalize on the small “window of opportunity”). Categorical moderator analyses will be conducted using the analog to the ANOVA method (see Lipsey & Wilson, 2001), while continuous moderators or analysis of multiple moderators simultaneously will be conducted using meta‐regression (see Higgins et al., 2020). Additional posthoc moderator analyses may also be conducted; however we will identify these as posthoc analyses in our final report.

### 3.19 Sensitivity analysis

In addition to separate analyses of randomized and quasi‐experimental studies and published and unpublished studies, sensitivity analyses may include separate analyses by study location (city, state, country), research team, and risk of bias ratings.

### 3.20 Treatment of qualitative research

We do not plan to include qualitative research in this review.

## ROLES AND RESPONSIBILITIES

- Content: Robert C. Davis, Kevin Petersen, David Weisburd, and Bruce Taylor.
- Systematic review methods: Robert C. Davis, Kevin Petersen, David Weisburd, and Bruce Taylor.
- Statistical analysis: Bruce Taylor and Kevin Petersen.
- Information retrieval: Robert C. Davis and Kevin Petersen.

## SOURCES OF SUPPORT

L 10,000 Incentive Award from the National Institute for Health Research.

## DECLARATIONS OF INTEREST

Robert C. Davis, Bruce Taylor, and David Weisburd produced the earlier review of second responder programs. Robert C. Davis and Bruce Taylor have also conducted three of the studies previously reviewed. Kevin Petersen has not conducted evaluation research or published on the effectiveness of second responder programs.

## PRELIMINARY TIMEFRAME

The review process will adhere to the following schedule:

**Table:** 

Submission of protocol	June 10, 2021
Completion of GPD search	July 31, 2021
Coding of eligible studies	August 1–15, 2021
Submission of completed report	October 1, 2021

## Supporting information

Supplementary Information

## Footnotes

1
https://thrivenyc.cityofnewyork.us/program/crime-victim-assistance-program-in-precincts-and-psas

2 By similar case make‐up, we mean that the distribution of types of offenses (e.g., assault, harassment, violation of restraining order, and so forth, had to be comparable between the intervention and control areas. By similar demographic make‐up, we mean that the distribution of basic demographics available on police reports (victim and abuser age, gender, and race) and from census data (indicators of concentrated disadvantage) had to be comparable between the intervention and control sites.
