# Supplementary material for: Updated protocol: Effects of second responder programs on repeat incidents of family abuse: An updated systematic review and meta‐analysis
Source: Campbell Syst Rev. 2021 Nov 10;17(4):e1200. doi: 10.1002/cl2.1200 (PMC8988767; doi:10.1002/cl2.1200)
Supplement: Supplementary file 1 — Supplementary Information [file CL2-17-e1200-s001.DOCX]

Appendix A: GPD Systematic Search Strategy^[[1]](#footnote-2)^

### Search Terms

To ensure optimum sensitivity and specificity, the GPD search strategy utilises a combination of free-text and controlled vocabulary search terms. Because controlled vocabularies and search capabilities vary across databases, the exact combination of search terms and field codes are adapted to each database. Final search syntax for each location will be reported in the final review.

The free-text search terms for the GPD are provided in Table 1 and are grouped by substantive (i.e., some form of policing) and evaluation terminology. Although the search strategy may vary slightly across search locations, it follows a number of general rules:

- Search terms are combined into search strings using Boolean operators “AND” and “OR”. Specifically, terms within each category are combined with “OR”, and categories will be combined with “AND”. For example: (police OR policing OR “law#enforcement”) AND (analy* OR ANCOVA OR ANOVA OR …).
- Compound terms (e.g., law enforcement) are considered single terms in search strings by using quotation marks (i.e., “law*enforcement”) to ensure that the database searches for the entire term rather than separate words.
- Wild cards and truncation codes are used for search terms with multiple iterations from a stem word (e.g., evaluation, evaluate) or spelling variations (e.g., evaluat* or randomi#e).
- If a database has a controlled vocabulary term that is equivalent to “POLICE”, the term is combined in a search string that includes both the policing and evaluation free-text search terms. This approach ensures that the search retrieves documents that do not use policing terms in the title/abstract but have been indexed as being related to policing in the database. An example of this approach is the following search string: (((SU: “POLICE”) OR (TI,AB,KW: police OR policing OR “law*enforcement”)) AND (TI,AB,KW: intervention* OR evaluat* OR compar* OR …)).
- For search locations with limited search functionality, a broad search that uses only the policing free-text terms is implemented.
- Multidisciplinary database searches are limited to relevant disciplines (e.g., include social sciences but exclude physical sciences).
- Search results are refined to exclude specific types of documents that are not suitable for systematic reviews (e.g., newspapers, front/back matter, book reviews).

#### Table 1. Free-text search terms for the GPD systematic search

| **Policing Search Terms** | **Evaluation Search Terms** | | | |
| --- | --- | --- | --- | --- |
| police  policing  “law*enforcement”  constab*  detective*  sheriff* | analy*  ANCOVA  ANOVA  “ABAB design”  “AB design”  baseline  causa*  “chi#square”  coefficient*  “comparison condition*”  “comparison group*”  “control condition*”  “control group*”  correlat*  covariat*  “cross#section*” | data  effect*  efficacy  eval*  experiment*  hypothes*  impact*  intervent*  interview*  longitudinal  MANCOVA  MANOVA  “matched group”  measure*  “meta-analy*”  “odds#ratio* | outcome*  paramet*  “post-test”  posttest  “post test”  predict*  “pre-test”  pretest  program*  “propensity score*”  quantitative  “quasi#experiment*”  questionnaire*  random*  RCT  regress* | result*  “risk#ratio*”  sampl*  “standard deviation*”  statistic*  studies  study  survey*  “systematic review*”  “t#test*”  “time#series”  treatment*  variable*  variance |

### Search Locations

To reduce publication and discipline bias, the GPD search strategy adopts an international scope and involves searching for literature across a number of disciplines (e.g., criminology, law, political science, public health, sociology, social science and social work). The search captures a comprehensive range of published (i.e., journal articles, book chapters, books) and unpublished literature (e.g., working papers, governmental reports, technical reports, conference proceedings, dissertations) by implementing a search strategy across bibliographic/academic, grey literature, and dissertation databases or repositories.

It is noted that there is substantial overlap of the content coverage between many of the databases. Therefore, the *Optimal Searching of Indexing Databases* (OSID) computer program (Neville & Higginson, 2014) has been used to analyse the content crossover for all databases that have accessible content coverage lists. OSID analyses the content coverage and creates a search location solution that provides the most comprehensive coverage via the least number of databases. Another advantage of using OSID when designing a search strategy is the reduction in the number of duplicates that would need to be removed prior to the screening phase. Databases with >10 unique titles are searched in full, whereas databases with ≤10 unique titles were searched only the unique titles and any non-serial content (e.g., reports, conference proceedings). Where a modified search of a database would be more labour-intensive than a full search and export results, a full search of the database is conducted. The final search locations and solutions are reported in Table 2.

#### Table 2. GPD search locations and protocol (January 1^st^ 1950 – December 2018)

| **INDEXED & ACADEMIC DATABASES** |  | **CONTENT COVERAGE FED INTO OSID?** | **FULL OR MODIFIED SEARCH?** | **SEARCH MODIFICATIONS** |
| --- | --- | --- | --- | --- |
| **ProQuest** | Criminal Justice | Yes | Full | None. |
|  | Dissertation and Theses Database Global | Not Available | Modified | Social Sciences subset. |
|  | Political Science | Yes | Full | None. |
|  | Periodical Archive Online | Yes | Full | None. |
|  | Research Library | Yes | Modified | Social Sciences subset. |
|  | Social Science Journals | Yes | Full | None. |
|  | Sociology | Yes | Modified | Search 2 unique journal titles and non-serial content only. |
|  | Applied Social Sciences Index and Abstracts | Yes | Full | None. |
|  | International Bibliography of the Social Sciences | Yes | Full | None. |
|  | Public Affairs Information Service | Yes | Full | None. |
|  | Social Services Abstracts | Yes | Modified | Search 5 unique journal titles and non-serial content only. |
|  | Sociological Abstracts | Yes | Full | None. |
|  | Worldwide Political Sciences Abstracts | Yes | Modified | Search 9 unique journal titles and non-serial content only. |
| **EBSCO** | Academic Search Premier | Yes | Full | None. |
|  | Criminal Justice Abstracts | Yes | Full | None. |
|  | EconLit | Yes | Full | None. |
|  | MEDLINE with Full-Text | Yes | Full | None. |
|  | Social Sciences Full-Text | Yes | Full | None. |
| **OVID** | International Political Science Abstracts | Not Available | Full | None. |
|  | PsycARTICLES | Yes | Modified | Search 4 unique journal titles only. |
|  | PsycEXTRA | Not Available | Full | None. |
|  | PsycINFO | Yes | Full | None. |
|  | Social Work Abstracts | Not Available | Full | None. |
| **Web of Science** | Current Contents Connect – Social and Behavioural Sciences Edition | Yes | Modified | Search 1 unique journal title and non-serial content only. |
|  | Book Citation Index (Social Sciences and Humanities) | Not Available | Full | None. |
|  | Conference Proceedings Citation Index (Social Sciences and Humanities) | Not Available | Full | None. |
|  | Social Science Citation Index | Yes | Full | None. |
| **Informit** | Australian Attorney General Information Service | Yes | Full | None. |
|  | Australian Criminology Database (CINCH) | Yes | Full | None. |
|  | Australian Federal Police Database | Yes | Full | None. |
|  | Australian Public Affairs Full-Text | Yes | Full | None. |
|  | DRUG | Yes | Full | None. |
|  | Health & Society Database | Yes | Modified | Search unique journal titles and non-serial content only. |
|  | Humanities and Social Sciences Collection | Yes | Full | None. |
| **Gale-Cengage** | Expanded Academic ASAP | Yes | Full | None. |
| **STANDALONE & OPEN ACCESS DATABASES** | Cambridge Journals Online | Yes | Modified | Search 4 unique journal titles in Law and Political Science collections and full search of Social Studies collection. |
|  | Directory of Open Access Journals | Yes | Full | None. |
|  | HeinOnline | Yes | Modified | Law Journals Online collection only. |
|  | JSTOR | Yes | Modified | Search unique titles across the Law, Political Science, Public Health, Public Policy, Social Work and Sociology collections only. The Criminal Justice collection had no unique content and so will be excluded from the search. Only 10% of content in this database have abstracts and a full-text search returns >250,000 results because of inability to construct complex search strings. Therefore, a modified search of the unique titles across these collections will be more pragmatic than a full search of the database. |
|  | Oxford Scholarship Online | Yes | Full | None. |
|  | Sage Journals Online and Archive (Sage Premier) | Yes | Modified | Search 5 unique journal titles and non-serial content only. |
|  | ScienceDirect | Yes | Full | None. |
|  | SCOPUS | Yes | Full | None. |
|  | SpringerLink | Yes | Full | Although this database has low uniqueness when combined with the full set of databases, a full search using only the policing search terms will be more pragmatic than a modified search on unique titles because of the restricted search functionality of this database. |
|  | Taylor & Francis Online | Yes | Modified | Although this database has low uniqueness when combined with the full set of databases, a full search using only the policing search terms will be more pragmatic than a modified search on unique titles because of the restricted search functionality of this database. |
|  | Wiley Online Library | Yes | Full | None. |
|  | California Commission on Peace Officer Standards & Training Library | No | Full | None. |
|  | Cochrane Library | No | Full | None. |
|  | CrimeSolutions.gov | No | Full | None. |
|  | Database of Abstracts of Reviews of Effectiveness (DARE) | No | Full | None. |
|  | FBI – The Fault (Reports and Publications) | No | Full | None. |
|  | Evidence-Based Policing Matrix | No | Full | None. |
|  | International Initiative for Impact Evaluation Database (3ie) | No | Full | None. |
|  | National Criminal Justice Reference Service | No | Full | None. |
|  | Safety Lit Database | No | Full | None. |
|  | Australian Institute of Criminology | No | Full | None. |
|  | Bureau of Police Research and Development (India) | No | Full | None. |
|  | Canadian Police Research Catalogue | No | Full | None. |
|  | Centre for Problem-Oriented Policing | No | Full | None. |
|  | College of Policing (including POLKA and Crime Reduction Toolkit) | No | Full | None. |
|  | European Police College (CEPOL) | No | Full | None. |
|  | Evidence for Policy and Practice Information and Coordinating Centre | No | Full | None. |
|  | National Research Institute of Police Science (Japanese) | No | Full | None. |
|  | Office of Community Oriented Policing Services | No | Full | None. |
|  | Police Executive Research Forum (US) | No | Full | None. |
|  | Police Foundation (US) | No | Full | None. |
|  | Tasmania Institute of Law Enforcement Studies (Australia) | No | Full | None. |
|  | Policing Online Information System (POLIS, Europe) | No | Full | None. |
|  | Scottish Institute for Policing Research | No | Full | None. |
|  | Centre of Excellence in Policing and Security (Australian, now archived) | No | Full | None. |

Appendix B: GPD Systematic Compilation Strategy

### Inclusion Criteria

Each record captured by the GPD systematic search must satisfy all inclusion criteria to be included in the GPD: timeframe, intervention and research design. There are no restrictions applied to the types of outcomes, participants, settings or languages considered eligible for inclusion in the GPD.

#### Types of interventions

Each document must contain an impact evaluation of a policing intervention. Policing interventions are defined as some kind of a strategy, program, technique, approach, activity, campaign, training, directive, or funding/organisational change that involves police in some way (other agencies or organisations can be involved). Police involvement is broadly defined as:

- Police initiation, development or leadership
- Police are recipients of the intervention or the intervention is related, focused or targeted to police practices
- Delivery or implementation of the intervention by police

#### Types of study designs

The GPD includes quantitative impact evaluations of policing interventions that utilise randomised experimental (e.g., RCTs) or quasi-experimental evaluation designs with a valid comparison group that does not receive the intervention. The GPD includes designs where the comparison group receives ‘business-as-usual’ policing, no intervention or an alternative intervention (treatment-treatment designs).

The specific list of research designs included in the GPD are as follows:

- Systematic reviews with or without meta-analyses
- Cross-over designs
- Cost-benefit analyses
- Regression discontinuity designs
- Designs using multivariate controls (e.g., multiple regression)
- Matched control group designs with or without pre-intervention baseline measures (propensity or statistically matched)
- Unmatched control group designs with pre-post intervention measures which allow for difference-in-difference analysis
- Unmatched control group designs without pre-intervention measures where the control group has face validity
- Short interrupted time-series designs with control group (less than 25 pre- and 25 post-intervention observations)
- Long interrupted time-series designs with or without a control group (≥25 pre- and post-intervention observations)
- Raw unadjusted correlational designs where the variation in the level of the intervention is compared to the variation in the level of the outcome

The GPD excludes single group designs with pre- and post-intervention measures as these designs are highly subject to bias and threats to internal validity.

### Systematic Screening

To establish eligibility, records captured by the GPD search progress through a series of systematic stages which are summarised in Figure 1, with additional detail provided in the following subsections.

All research staff working on the GPD undergo standardised training before beginning work within any of the stages detailed below. Staff then complete short training simulations to enable an assessment of their understanding of the GPD protocols and highlight any areas for additional training. In addition, random samples of each staff’s work are regularly cross-checked to ensure adherence to protocols. Disagreements about screening decisions between staff are mediated by either the project manager or GPD chief investigators.

#### Title and abstract screening

After removing duplicates, the title and abstract of records captured by the GPD systematic search is screened by trained research staff to identify potentially eligible research that satisfies the following criteria:

- Document is dated between 1950 – present
- Document is unique (i.e., not a duplicate)
- Document is about police or policing
- Document is an eligible document type (e.g., not a book review)

Records are excluded if the answer to any one of the criteria is unambiguously ‘No’, and will be classified as potentially eligible otherwise. Records classified as eligible at the title and abstract screening stage progress to full-text document retrieval and screening stages.

#### Full-text eligibility screening

Wherever possible, a full-text electronic version of an eligible record is imported into *SysReview* (review management software; Neville & Higginson, 2014). For records without an electronic version, a hardcopy of the record is located to enable full-text eligibility screening. The full-text of each document is screened to identify studies that satisfy the following criteria:

- Document is dated between 1950 – present
- Document is unique
- Document reports a quantitative statistical comparison
- Document reports on policing evaluation
- Document reports in a quantitative impact evaluation of a policing intervention
- Evaluation uses an eligible research design

| **SYSTEMATIC SEARCH OF PUBLISHED & UNPUBLISHED LITERATURE** |
| --- |
|  |
| **EXPORT SEARCH RESULTS**   - Bibliographic data and abstracts exported into EndNote - Data cleaned and duplicate records removed |
|  |
| **IMPORT SEARCH RESULTS INTO *SYSREVIEW*** |
|  |
| **SCREEN TITLES AND ABSTRACTS FOR ELIGIBILITY**   1. Not a duplicate document? 2. Between 1950 – present? 3. About police or policing? 4. Eligible document type?   ***If not clearly excluded on any criteria…*** |
|  |
| **DOCUMENT RETRIEVAL**   - Retrieve electronic and hard copies of all eligible documents - Attach electronic versions to records in *SysReview* |
|  |
| **SCREEN FULL-TEXT OF DOCUMENTS**  **FOR FINAL ELIGIBILITY**   1. Not a duplicate document? 2. Between 1950 – present? 3. Quantitative statistical comparison? 4. Policing intervention? 5. Quantitative impact evaluation? 6. Eligible research design?   ***If ‘Yes’ to all…*** |
|  |
| **CATEGORISE ELIGIBLE DOCUMENTS**   1. Research design 2. Intervention location 3. Publication date 4. Problem targeted 5. Evaluation outcome measure(s) 6. Type of policing intervention |
|  |
| **GLOBAL POLICING DATABASE (GPD)**  Web-based  Searchable  Updated biennially |

*Figure 1.* GPD systematic compilation process

Appendix C: Second responders meta-analysis coding sheet

**I. ELIGIBILITY CHECK SHEET**

1. Document ID: __ __ __ __

2. First author last name:________________

3. Study Title:____________________________

4. Journal Name, Volume and Issue: _______________________________________

5. Document ID: __ __ __ __

6. Coder’s Initials __ __ __

7. Date eligibility determined: ____________

8. A study must meet the following criteria in order to be eligible. Answer each question with a “yes” or a “no”

a. Study is an evaluation of a second responder program, that is a program operated by or in cooperation with a municipal law enforcement agency that attempts to visit the homes of victims following the initial police response to a family violence incident. _____

b. Study includes a comparison group which did not receive a second response. ______

c. The study reports on at least one measure of repeat family violence crimes and/or abuse derived from police reports or surveys with victims.

. ______

d. The study is written in English. _____

If the study does not meet the criteria above, answer the following question:

a. The study is a review article that is relevant to this project (e.g. may have references to other studies that are useful, may have pertinent background information) ______

9. Eligibility status:

____ Eligible

____ Not eligible

____ Relevant review

Notes: ____________________________________________________________________________________________________________________________________________________________

**II. CODING PROTOCAL**

**Reference Information**

1. Document ID: __ __ __ __

2. Study author(s): ____________________

3. Study title: _______________________

4a. Publication type: ______

1. Book

2. Book chapter

3. Journal article (peer reviewed)

4. Thesis or doctoral dissertation

5. Government report (state/local)

6. Government report (federal)

7. Police department report

8. Technical report

9. Conference paper

10. Other (specify)

4b. Specify (Other)_____________________

5. Publication date (year): ______________

6a. Journal Name: ____________________

6b. Journal Volume: _______________

6c. Journal Issue: ____________

7. Date range of research (when research was conducted):

Start: ____________

Finish: ____________

8. Source of funding for study: ___________________

9. Country of publication: ___________________

10. Date coded: ___________

11. Coder’s Initials: __ __ __

**Describing the Sample**

12. What types of incidents were eligible? (Select all that apply)

1. Intimate partner cases

2. Family abuse cases

3. Elder abuse cases

4. Other (specify)

12b. Specify (Other) _____________

13. What kinds of criminal charges were eligible? (Select all that apply)

1. Assault

2. Harassment

3. Menacing

4. Violation of restraining order

5. Other (specify)

13b. Specify (Other) _____________

14. What type police responses were eligible?

1. Cases in which an arrest was made

2. Cases in which crime complaints were filed

3. Any report, founded or unfounded

4. Other (specify)

14b. Specify (Other) ___________

14c. What recruitment method was used to select cases?

1. Assignment of official reports/calls for service

2. Assignment by volunteer status

3. Other

14d. Specify (Other) ______________

15**.** Other than the factors described in 12-14, describe any restrictions on selecting cases for the sample: Is there reason to think that the sample is not representative of all DV complaints within the jurisdiction studied?

16. Characteristics of victims in the sample (enter N/I for not included)

a. Average age _______

b. Gender

1. Percent female ________ %

2. Percent male _________ %

c. Education

1. High school grad _______ %

2. Did not graduate high school ______ %

d. Currently employed

1. Employed full time _____ %

2. Employed part-time _____ %

3. Not employed ______ %

e. Residence type

1. Own home ______ %

2. Rent _______ %

3. Public housing _______ %

17. Characteristics of perpetrators in the sample (enter N/I for not included)

a. Average age _______

b. Gender

1. Percent female ________ %

2. Percent male _________ %

c. Education

1. High school grad _______ %

2. Did not graduate high school ______ %

d. Currently employed

1. Employed full time _____ %

2. Employed part-time _____ %

3. Not employed ______ %

e. Residence type

1. Own home ______ %

2. Rent _______ %

3. Public housing _______ %

**Describing the Response**

18. What did home visits consist of? (Select all that apply)

1. Assess victim’s current situation and history of abuse in relationship

2. Develop safety plan with victim

3. Discuss nature of abuse

4. Assess victim needs

5. Provide information and referrals to service programs

6. Interact with abuser

7. Other

18b. Specify (Other)___________________

19. Who was involved in the implementation of the response? (Select all that apply)

1. Domestic/family violence police officer

2. Police victim caseworker

3. Prosecutor victim caseworker

4. Independent victim advocate

5. Other (specify)

19b. Specify (Other)___________________

20. How long after the incident was reported was the second response attempted?

1. Within 24 hours

2. Within several days of incident

3. More than several days after incident

4. Other (specify)

20b. Specify (Other)___________________

21c. If provided, what was the average number of hours that elapsed between the incident and the second response attempt? ________________

21**.** Is information provided on the average length of visits?

1. No

2. Yes 🡺 Average length: ______________________________

22. Was the visit unannounced, or was there an attempt made to call the victim first?

1. Visits were typically unannounced

2. Phone contact attempted prior to visit

3. Other (specify)

23. Is information available on how often perpetrators were present during visit?

1. No

2. Yes 🡺 % of cases: ________________________________

23b. Specify (Other)___________________

24. Did the second response program exist prior to the evaluation, or was program implemented in conjunction with the evaluation?

1. Program implemented in conjunction with evaluation

2. Program existed prior to evaluation 🡺 For how long? __________________

25. Does study indicate that author(s) had a relationship with the program prior to the evaluation?

1. No indication of prior relationship

2. Paper indicates prior relationship (describe: ___________________________)

***Implementation of Response***

26. In what proportion of targeted households did the second responders establish face-to-face contact with the victim? _____ %

27. If face-to-face contact could not be established, what did the intervention consist of (e.g., literature or letter left; phone call):

________________________________________________________________________________________________________________________________________________________________________________________________________________________

***Location of the intervention***

28. Country where study was conducted: __________________

29. City (and state/province, if applicable) where study was conducted: _________________

*The following questions refer to the area receiving treatment:*

30. Geographic area receiving treatment: ______

1. Micro place (street segments/blocks)/ public housing development

2. Neighborhood/police beat

3. Police district/precinct

4. Entire city

5. Other (specify)

30b. Specify (Other)___________________

31. What is the exact geographic area receiving treatment? ________________________________________________________________________

*The following refer to the area not receiving treatment (applicable if there is a separate control group in the study)*

32. Was comparison group drawn from different geographic area than treatment group?

1. No ==> *Skip to Q 35*

2. Yes

33a. Geographic area NOT receiving treatment: ______

1. Micro place (street segments/blocks)/public housing development

2. Neighborhood/police beat

3. Police district/precinct

4. Entire city

5. Other (specify)

6. N/A (no control area)

33b. Specify (Other)___________________

34. What is the exact geographic area not receiving treatment? ________________________________________________________________________

***Confounding interventions***

35. Was the second response treatment confounded with any other interventions (e.g., enhanced evidence collection or prosecution)? Describe: _________________________

**Methodology/Research design:**

36. Type of study:

1. Randomized experiment ==>

36a. How were cases randomized?___________________________

____________________________________________________

36b. What was the rate and cause of experimental misassignments? _______________ ___________________________________

___________________________________________________

36c. Were misassignments analyzed as assigned or as treated?

1. Analyzed as assigned
2. Analyzed as treated
3. Nonequivalent control group (quasi-experimental) ==> 30d. How were

control cases selected?

1. Matched cases (method of matching:_________________________)
2. According to objective case criteria (specify:__________________)
3. Based on possibly unbiased variable (e.g., time of day, precinct)
4. Selected by staff conducting intervention using subjective criteria
5. Treatment refusers or drop-outs
6. Other (Specify: _________________________________________)

37. If more than one treatment or comparison group used, describe nature of each:

Treatment groups Comparison groups

38. Were efforts made to determine similarity in case, victim, and/or perpetrator criteria between treatment and comparison groups?

1. No

2. Yes 🡺 31a. What were the results? _________________________________

____________________________________________________________

____________________________________________________________

**39.** Did researchers believe any baseline differences biased study results? If so, in what direction? _______________________________________________________________

***Outcomes reported***

40. Which outcome measures are reported in the study?

Same victim as Any

original incident victim

1. New family violence incident reports to the police ____ _____

2. New family violence arrests ____ _____

3. New arrests for any offense ____ _____

4. New abuse (from victim survey)

4. Satisfaction with police response (from victim survey)

5. Willingness to report future incidents (from victim survey)

4. Awareness/use of victim services (from victim survey)

40b. Specify (other) _________

**NOTE: COMPLETE ITEMS 41-60 FOR EACH OUTCOME MEASURE REPORTED (AND FOR EACH SET OF CONTROL-TREATMENT COMPARISONS, IF MORE THAN ONE CONTROL OR TREATMENT GROUP)**

41. If victim surveys were used, what was response rate? ________ %

42. Did the researcher assess the quality of the data collected?

1. Yes

2. No

43. Did the researcher(s) express any concerns over the quality of the data?

1. Yes

2. No

43b. If yes, explain ____________________________________________________________________________________________________________________________________________________________

**Effect size/Reports of statistical significance**

*Dependent Measure Descriptors*

***Sample size***

44. What is the total sample size in the analysis? ________

45. What is the total sample size of the treatment group (group that receives the response)? _______

46. What is the total sample size of the control group? _____

47. Did the analysis include all cases assigned to treatment or only those with whom face-to-face contact was established by the home visit team?

1. All cases assigned to treatment

2. Only cases where victim was home at time of visit

***Effect Size Data***

48. Raw difference favors (i.e. shows more success for):

1. Treatment group

2. Control group

3. Neither (exactly equal)

9. Cannot tell (or statistically insignificant report only)

49. Did a test of statistical significance indicate statistically significant differences between the control and treatment groups?

1. Yes

2. No

3. Can’t tell

4. N/A (no testing completed)

50. Was a standardized effect size reported?

1. Yes

2. No

51. If yes, what was the effect size? ______

52. If yes, page number where effect size data is found ________

53. If no, is there data available to calculate an effect size?

1. Yes

2. No

54. Type of data effect size can be calculated from:

1. Means and standard deviations

2. *t*-value or *F*-value

3. Chi-square (df=1)

4. Frequencies or proportions (dichotomous)

5. Frequencies or proportions (polychotomous)

6. Other (specify)

54b. Specify (other) _________

*Means and Standard Deviations*

55a. Treatment group mean. _____

55b. Control group mean. _____

56a. Treatment group standard deviation. _____

56b. Control group standard deviation. _____

*Proportions or frequencies*

57a. *n* of treatment group with a successful outcome. _____

57b. *n* of control group with a successful outcome. _____

58a. Proportion of treatment group with a successful outcome. _____

58b. Proportion of control group with a successful outcome. _____

*Significance Tests*

59a. *t*-value _____

59b. *F*-value _____

59c. Chi-square value (*df*=1) _____

*Calculated Effect Size*

60. Effect size ______

**Conclusions made by the author(s)**

61. Conclusion about the impact of the intervention?

1. The authors conclude abuse declined

2. The authors conclude abuse did not decline

3. Unclear/no conclusion stated by authors

62. Did the author(s) conclude that the second responder intervention was beneficial?

1. Yes

2. No

3. Can’t tell

63. Did the author(s) conclude there a relationship between the treatment and a reduction in abuse? _____

1. Yes

2. No

3. Can’t tell

64. Additional notes about conclusions:

____________________________________________________________________________________________________________________________________________________________

1. Appendices A and B are taken directly from Higginson, A., Eggins, E., Mazerolle, L. and Stanko, E. (2015). *The Global Policing Database [Database and Protocol].* [↑](#footnote-ref-2)
